# Four-dimensional metal-organic frameworks

**DOI:** 10.1038/s41467-020-16527-8

**Published:** 2020-06-01

**Authors:** Jack D. Evans, Volodymyr Bon, Irena Senkovska, Hui-Chun Lee, Stefan Kaskel

**Affiliations:** Technische Universität Dresden, Bergstrasse 66, 01062 Dresden, Germany

**Keywords:** Metal-organic frameworks, Soft materials, Metamaterials, Characterization and analytical techniques

## Abstract

Recognising timescale as an adjustable dimension in porous solids provides a new perspective to develop novel four-dimensional framework materials. The deliberate design of three-dimensional porous framework architectures is a developed field; however, the understanding of dynamics in open frameworks leaves a number of key questions unanswered: What factors determine the spatiotemporal evolution of deformable networks? Can we deliberately engineer the response of dynamic materials along a time-axis? How can we engineer energy barriers for the selective recognition of molecules? Answering these questions will require significant methodological development to understand structural dynamics across a range of time and length scales.

## Abstract (web summary)

As the field of metal-organic frameworks is maturing, understanding the dynamics of open frameworks is progressing and rational approaches are under development. Here, the authors outline challenges and potential routes to engineering the spatio-temporal evolution of dynamic metal-organic frameworks.

## Introduction

Porous framework materials offer outstanding functionality due to their wide range of tuneable building blocks and ultrahigh porosity^1–5^. The integration of functions, such as chemisorptive, redox, optically or catalytically active centers, encapsulated drugs, enzymes, nanoparticles, and more, has led to the discovery of metal-organic framework (MOF) applications in gas separation, CO_2_ sequestration, gas storage, catalysis, optics, and sensing, and a roadmap for integration into electronic systems has even been proposed^6–12^.

The chemistry of three-dimensional (3D) porous framework materials, connecting nodes and linkers through a variety of chemical bonds is enormously rich^13,14^. While decades ago, rationalization was aided by topology analysis and isoreticular expansion^15,16^, in the age of digitalization computer-aided design plays a crucial role in predicting millions of new 3D structures and their properties^17–19^. The serendipitous discovery of an adsorption induced structural transformation in a network nowadays termed ELM-11^20^ stimulated researchers and laid the foundations for exploring novel dynamic phenomena in porous frameworks. Like enzymes, structural changes are stimulated by guest molecules intruding into the porous frameworks leading to macroscopic volume changes of up to 200–300%. The adaptive change of pore size is a fascinating feature, but a selectivity comparable to that of enzymes has not been reached yet.

## Emerging applications of adaptive porous materials

**Fig. 1:**
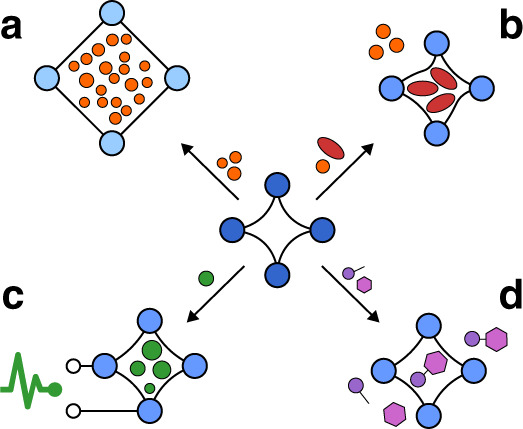
The prominent functions of dynamic MOFs. **a** Ideal deliverable capacity in gas storage^28^. **b** Energy efficient highly selective separation^129^. **c** Selective sensing^40^. **d** Switchable catalysis^48^.

Porosity switching in the crystalline solid state represents a unique phenomenon observed only in a limited number of porous materials^21–25^. This flexibility was predicted in 1998 for MOFs by Kitagawa and coworkers, and later termed “3^rd^ Generation MOFs”^25–27^. These materials are characterized by dynamic features of the framework structure and are also named “soft porous crystals” (SPCs). We briefly account here a few remarkable applications of the dynamic frameworks reported today as the premise for our following perspective (Fig. 1). In gas storage applications, the pore closing upon desorption provides almost ideal deliverable capacity, a key advantage compared with rigid adsorbents^28^. More importantly, for gas separations, the selective response of the porous host in adapting the pore shape to a recognized gas molecule species leads to a significant increase in adsorption selectivity as compared to traditional rigid adsorbents^29^. For example, Long and colleagues demonstrated “near-perfect” CO_2_/CH_4_ selectivity, based on a size exclusion mechanism, using the flexible MOF Co(bdp) (bdp = 1,4-benzenedipyrazolate)^30^. Another interesting separation example for removal of propyne from propylene was reported for ELM-12^29^. A high propylene purity of over 99.9998% in experimental breakthrough curves for a 1/99 propyne/propylene mixture could be reached. The specific recognition in this case was also demonstrated using neutron scattering and theoretical calculations^29^. A similar flexible MOF, (Cu(dhbc)_2_(bpy); dhbc = 2,5-dihydroxybenzoate, bpy = 4,4′-bipyridine), which displays gate-opening characteristics, exhibits adsorption selectivity in favor of propyne for a C1-C3 hydrocarbon mixture^31^. A flexible MOF (UTSA-300), which transforms into a closed pore state after solvent removal, is able to efficiently separate C_2_H_2_ from CO_2_ and C_2_H_4_^32^. Warren et al. also reported the selective sorption of *para-* vs. *meta-*xylene by a flexible MOF^33^. One may envision, in the near future, the emergence of materials with the ability to open their pores only for one type of molecular species resulting in specific recognition. A spectacular example is the selective recognition of CO vs. N_2_ in a dynamic MOF, enabling the adsorptive separation of two gases, which have quite similar physical characteristics^34^. Notably, adsorptive separations play a key role in reducing CO_2_ emissions as demanding distillation processes account for 10–15% of the world energy consumption^35^.

The selective recognition of small molecules, and associated step-wise changes of physical properties (optical, electrical, and magnetic), enables the design of highly selective sensor concepts^36–39^. MOFs may function as a threshold sensor indicating the presence of toxic volatile compounds when a certain concentration level is surpassed^40,41^. Optically induced structural switching, and the change of optical properties as a response towards structural transformations, is ideally suited for fundamental time-resolved studies and might lead to membranes with photoswitchable selectivity^42–44^. A unique application is the electric field induced switching of gas permeation in a MOF membrane^45^. Switchable magnetic and electronic properties are also emerging for, in particular, information processing technologies^12,46,47^. One visionary application is a switchable catalyst, a catalytically active MOF exposing its active sites during opening of the porous system^48^. Even a dynamic framework, where the catalytic functionality is a direct consequence of dynamic adaption of the host structure and active sites are exposed for certain substrates, may be envisioned^49^. The huge volume change, exhibited by some dynamic MOFs, renders these materials as promising actuators^50^. Their response to environmental changes, such as humidity or other gases, is promising for the fabrication of autonomous soft materials. However, these examples appear relatively simple compared with the power and complex recognition mechanisms of biological macromolecules and their dynamic recognition of substrates, leading to highly specific binding and transformations. These underlying complex sequences of molecular building blocks and secondary folding mechanisms were genetically optimized during evolution providing unrivalled catalytic performance.

The modular construction of many frameworks in principle may allow for comparably complex architectures. Multivariate MOFs with up to eight different linkers were produced, leading mostly to statistical distribution, so far, but in some cases this distribution is quite precisely positioned by sequential linker installation (SLI)^51–54^. The integration of complex hydrogen bonding schemes has already led to highly selective MOF-based sorbents^49^. However, the fundamental understanding of dynamic frameworks is still at an early stage. Further advancement of basic knowledge will form an essential basis to develop this highly potent technology platform to maturity.

## Current understanding of dynamic phenomena in metal-organic frameworks

The mechanistic investigation of novel dynamic phenomena in MOFs is supported by a wide range of advanced in situ characterization methods explored in recent years, including, but not limited to, diffraction, scattering, vibrational, and nuclear magnetic resonance (NMR) spectroscopies and many more. This has opened our eyes to a new world of dynamic phenomena in soft frameworks. The general term “dynamic” includes a wide range of dissimilar phenomena and is not precise. A more systematic nomenclature is still to be developed.

In the following we briefly outline the hierarchy of framework motions and their underlying mechanisms as they are understood today.

All solids demonstrate collective vibrations (phonons), elastic thermally driven deformations. Characteristic phonons can indicate structural instabilities, particular for materials with low bulk and shear moduli typical for MOFs. High porosity facilitates thermal phonon excitation, frequently leading to negative thermal expansion^55,56^. Phonon energetics heavily depend on guest loading but phonon-mediated thermal expansion phenomena are more or less continuous and do not necessarily lead to step-wise structural transformations.

Truly novel phenomena and characteristics arise from step-wise (discontinuous) framework transformations in the solid state induced by, and hence coupled, to inclusion, chemi-, or physisorption phenomena occurring inside the extended pore system. Precisely these materials may be termed switchable as the transformations are step-wise, in contrast to continuous swelling processes observed in many polymers. Novel functionality arises from this pronounced structural switching of the host pore structure between an open (*op*) and a closed or narrow pore structure (*cp*, *np*), which encompass changes of pore size, geometry, orientation and/or exposure of functional groups, and metal centers. The solid-state transitions of the framework are now coupled to the kinetics and thermodynamics of guest inclusion (adsorption) leading to complex and hitherto unexpected adsorption isotherms.

Currently one may distinguish conceptually two different groups of phenomena:

### Case 1

**Fig. 2:**
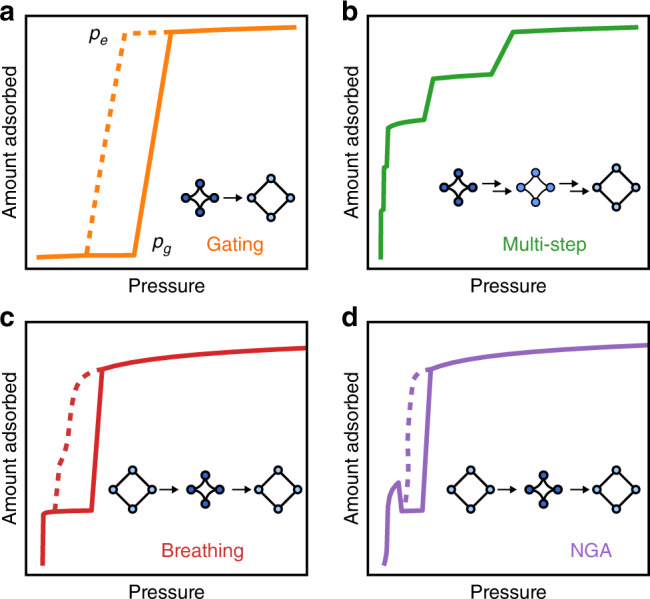
Dynamic adsorption phenomena in metal-organic frameworks. **a** Case 1a, gating (gate opening). **b** Case 1b, multistep opening. **c** Case 2a, breathing. **d** Case 2b, negative gas adsorption (NGA). Adsorption is depicted by solid lines and desorption dashed lines.

The conceptually most simple transformation pathway is most frequently termed gate opening (one step opening, case 1a, Fig. 2: closed pore, *cp* to open pore, *op*). In this case *cp* is energetically below *op* (*F*_*cp*_ < *F*_*op*_, *F* = Helmholtz free-energy of the empty host)^57^. Gate opening occurs if the guest stabilizes the *op* form, after reaching a system-specific activity (gate pressure, *p*_*g*_): *F(guest)*_*op*_ < *F*_*cp*_ for *p* > *p*_*g*_ (Fig. 2). It should be noted that *p*_*g*_ typically does not represent the equilibrium pressure (*p*_*e*_*, F(guest)*_*op*_ = *F*_*cp*_) for the *cp–op* transition, but the chemical potential necessary to overcome the activation barrier for pore opening (*p*_*g*_ > *p*_*e*_, *p*_*g*_ *−* *p*_*e*_ = Δ*p*)^58^. However, during desorption, the sigmoidal-shaped step may represent *p*_*e*_. For this guest-pressure-induced isothermal gate-opening transition *p* (or more general the guest activity, *a*) counteracts the elastic contribution of the barrier. This “overpressure” (Δ*p*) required to overcome a certain activation barrier plays a role analogous to the undercooling Δ*T* required to activate a thermally driven phase transition. In a thermally induced phase transition the change in volume Gibbs free-energy (Δ*G*_*V*_) is typically linear in undercooling Δ*T* = *T*_*e*_ *−* *T*, where *T*_*e*_ = equilibrium phase transition temperature, *L* = latent heat of transformation.

1 $${\mathrm{\Delta }}G_V \cong \frac{{ - L\Delta T}}{{T_e}}$$

Both, Δ*T* and Δ*p* reflect kinetic barriers for these dynamic transformations.

The gate opening (more precisely: *F*_*cp*_ < *F*_*op*_) case may in theory also lead to multistep adsorption isotherms (case 1b), in particular when layering or packing phenomena stabilize intermediate loading leading to one or multiple additional plateaus in the isotherm^59^.

### Case 2

Breathing phenomena instead result if *F*_*op*_ < *F*_*np*_^57,60–64^. The empty *op* host is more stable but host-guest interactions stabilize a narrow pore (*np*, sometimes also termed *cp* for contracted) phase, with reduced but non-zero porosity, at intermediate relative pressure. This intermediate stabilization is driven by the enhanced adsorption enthalpy, −Δ_ads_ *H*, of the guest for the *np* host ($|\Delta _{{\mathrm{ads}}}H_{np}| > |\Delta _{{\mathrm{ads}}}H_{op}|$). At full loading (*p/p*_*0*_ approaching 100 %) the higher pore volume of the *op* host can accommodate more guest molecules (*n*_ads*,op*_ > *n*_ads*,np*_) leading to a guest-stabilized *op* host (because $|\Delta _{{\mathrm{ads}}}H_{op} \cdot n_{{\mathrm{ads}},op}| \, > \, |\Delta _{{\mathrm{ads}}}H_{np} \cdot n_{{\mathrm{ads}},np}|$). The consequence is an *op–np–op* structural transition trajectory, following increasing relative pressure along the adsorption isotherm. Both, microporous (case 2a) and mesoporous materials (case 2b) show pronounced hysteresis in the isotherm, indicating one or both isotherm branches are controlled by activated cooperative processes. However, mesoporous systems may give rise to novel counterintuitive phenomena such as negative gas adsorption (NGA) (Fig. 2). In this case the first contraction (*op–np*) has a high activation barrier (the underlying principles here are yet to be fully uncovered) leading to a material that pushes out previously adsorbed guest molecules and an overall pressure amplification along the adsorption branch of the isotherm^65–69^.

## The open challenge of understanding frameworks spatiotemporal evolution

Despite several excellent reviews covering recent progress in dynamic framework materials, the fundamental understanding of inelastic dynamic transformations in open frameworks is in its infancy. In particular, deliberate tuning of the activation barriers involved poses enormous promise for engineering the kinetically controlled response behavior of dynamic MOFs. The intrinsic timescale of such responsive inelastic dynamic transformations is largely dominated by energetic barriers and metastable states, which are so far mostly inaccessible both from experimental and theoretical points of view. These energetic barriers may originate from: (1) the host structure being of molecular origin (bending of linkers, hinges at clusters), (2) cooperative phenomena (crystal size, domain size, and nucleation phenomena), and (3) the guest phase entering and condensing in the pore (transport into and in between pores, phase transitions of the fluid phase inside, nucleation phenomena, etc.).

The understanding and control of these activation barriers in switchable materials poses enormous potential for developing kinetically controlled frameworks. Controlling this intrinsic timescale of dynamic transformations in MOFs, we term as time-axis design (or short: *t*-axis design). We consider the time-axis as the 4th dimension, and term such MOFs with controlled and engineered energetic transformation barriers four-dimensional MOFs (or “4D-MOFs”). This term is clearly distinct from the “fourth generation porous materials (4 G)” introduced by Kitagawa and coworkers, which refers to a more comprehensive design of microstructures, such as hierarchy, hybrid composition, anisotropy, asymmetry, disorder, and defects, leading to complex functionality^70,71^. However, this term is rather broad and does not include a clear definition of a spatiotemporal evolution.

Instead, in our definition, we strictly refer to “4D” as addressing the temporal evolution as the 4th dimension of a dynamic material (the *t*-axis). This emphasizes the hitherto rarely explored dimension of “time” in dynamic frameworks. We define a “4D-MOF” as a framework for which the observer has characterized its spatiotemporal evolution, and can clearly represent it. The identification of various spatiotemporal functions (monotonic, periodic, etc.), and their representation, will ultimately prompt the deliberate engineering of materials with predefined *t*-axis. These materials, with deliberately tuned spatiotemporal evolution, might be then truly termed “designed” or “engineered” 4D-MOFs. This concept has tremendous implications and chances for exploration leading to a paradigm shift in functional materials chemistry.

## Towards 4D-MOFs as a new paradigm for materials design

**Fig. 3:**
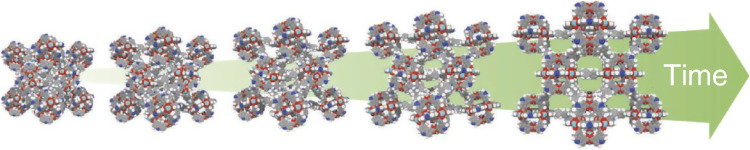
Four-dimensional MOFs. Engineering the spatiotemporal response of switchable metal-organic frameworks such as DUT-49^65^ along a time axis is in many respects an open field.

Dynamic phenomena are fascinating and movements are an essential characteristic of living creatures. If we could engineer energetic barriers of dynamic frameworks we could predetermine the transformation rate of such solids. To pose this central question in a simplified way: Can we synthesize a specific framework architecture as a fast or slow switching system? As a specific example, can we synthesize fast vs. slow switching porous frameworks with colossal stimuli-induced volume changes, such as DUT-49 (Fig. 3), or even predetermine their spatiotemporal evolution by means of constituents, microstructure, defect concentration, or specific molecular stimuli?

Is it even possible to achieve more complex movements in terms of the temporal evolution (or *t*-axis), including periodic oscillations, waves, walking, running, swinging, acceleration, damping, or even self-termination (collapse) after an “internal clock” has expired? Is it possible to deliberately program a material with an aperiodic movement along a temporal trajectory, in which the stimulus is only initiating the movement? Hence the *t*-axis is material-intrinsic and not artificially imposed by a stimulus varying in time. In other words: is it possible to engineer the spatiotemporal response of materials and can we deliberately tune the kinetics of structural transformations? It would be fascinating to make framework crystals jump fast, while others crawl slow. Can we implement a “time gene” into porous frameworks which determines their eventual fate?

These questions are highly relevant for dynamic materials but remain unanswered so far. Porous frameworks seem to be the ideal model materials for such a development due to their modular character and multivariate construction principles^51,52^. These developments may significantly profit from advanced techniques and understanding of spatiotemporal phenomena in catalysis^72–74^.

This vision of *t*-axis engineered materials is within reach and might have quite useful implications. As a simple example, the mechanical energy storage in dynamic MOFs has been proposed for dampers^75,76^. The necessity for controlling this kinetic response is a logical implication and a requirement to produce actuators/dampers with predefined response time. 4D-printing has been proposed for the generation of hydrogel based architectures and objects with dynamic self-control^77^. They mostly employ polymer swelling, or contraction, in response to contact with liquids and their dynamics are controlled by diffusion of the fluid phase. 4D-MOFs, however, are not limited in their response to liquids but offer a wide range of responses to gases. Moreover, their step-wise transformations will enable rapid movements and complex anisotropic motions.

**Fig. 4:**
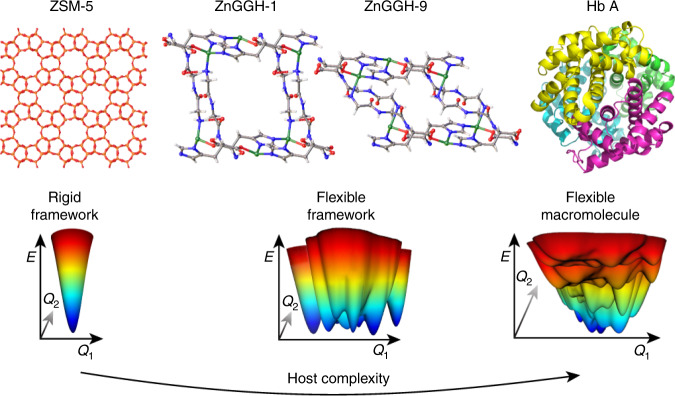
The potential landscape of advanced dynamic materials. Materials such as ZnGGH-1 and -9 compared to a flexible biological macromolecule, human haemoglobin (HbA) posing a higher degree of complexity as compared to rigid materials (zeolite ZSM-5). Reprinted by permission from Springer Nature, ref. ^49^.

The engineering of energetic barriers in MOFs is highly challenging. However, subtle differences in barriers associated with recognition of molecules differing slightly in structure will enable highly selective recognition and enzyme-like transformations. A remarkable achievement in this direction was recently reported for a MOF with hydrogen bonding network interactions controlling highly selective guest uptake (Fig. 4)^49^. The peptide constituents, and their interactions, result in a highly complex energetic landscape involving subtle minima and activation barriers providing high selectivity and control of the dynamic response towards a specific guest. In such complex systems, the frameworks free-energy landscape is, by itself, a highly dynamic recording of the history of adaptive guest inclusion evolving in time.

“Time” represents a hitherto underdeveloped perspective of dynamic MOFs. Recognizing this 4th dimension in switchable MOFs requires a paradigm shift away from the traditional static view of solid-state crystal structures. Such efforts first and foremost require the development of advanced analytical tools for “viewing” but also a development of new language for materials with a “history”.

For this perspective, we formulate four central and open questions towards 4D porous frameworks:

1. What are the intrinsic timescales of inelastic structural transformations*?*
2. How should we rationalize them (including language, theoretical concepts, and computational methods)?
3. What tools are available for analyzing the *t*-axis of dynamic MOFs and what methodological developments are necessary?
4. Can we deliberately design the temporal evolution of dynamic frameworks for truly 4D engineering?

This deliberate engineering of solid-state transformation rates would ultimately lead to a new generation of porous solids, with rationally adjusted local and collective barriers. The simplest version is comparable to a preprogrammed wind-up toy. A highly advanced 4D-MOF will contain precise structural sequence information like a “time-gene” that predetermines the temporal evolution of the material.

## The temporal dimension of dynamic framework phenomena

Static 3D architectures of frameworks are well understood, in terms of energetics, and structural prediction is routine using density functional theory (DFT), classic potentials and other methods. However, prediction of activation energies and transition rates for dynamic frameworks is at an early stage. Beyond the need for powerful computational methods, fundamental complexity arises from the timescale coupling of dissimilar phases, namely the framework (solid) and the molecular stimulus (often a fluid). The intrinsic timescale for dynamic framework deformations, as a phase transition proceeding with a typical phonon propagation rate may be expected roughly in the order of 10^−9^ s, with a characteristic temperature dependence. The temperature and pressure dependence of phase transitions, however, is highly non-linear and a few degrees of undercooling can increase nucleation rates by several orders of magnitude.

Alternatively, the molecular diffusion timescale, required for guest molecules to enter a porous framework crystal, varies in a wide range (10^−3^–10^4^ s) depending on pore size, crystal dimensions, temperature, pressure etc. The self-diffusion coefficient *D* relates the molecular mean-squared displacements *<r*^*2*^*>* to the observation time *t* via Einstein’s relation (*<r*^*2*^*>* = *6 Dt*). Moreover, nucleation of the fluid in a confined space may be delayed depending on pore size and distance from the critical point, imposing a second dimension of history-dependent states of the adsorbate^78^. Additional material-intrinsic timescales may also arise from cooperativity effects and defects dominating the size effects and metastability recently observed^66,79,80^.

Before we can engineer open frameworks in 4D, a fundamental understanding of the full free-energy landscape, as illustrated in Fig. 4, is required. Advanced dynamic materials providing functionality through complex hydrogen bonding architectures are intrinsically connected to complex free-energy landscapes and fine tuning of activation energies resulting in deliberately engineered kinetic pathways (Fig. 4). Exploring the timescale of these dynamic deformations is in many respects an open field.

Temporal changes are ultimately connected to non-equilibrium. Hence, observations of metastable intermediates mark early examples of 4D frameworks. A prominent example is the “shape memory effect” observed in Cu_2_(bdc)_2_bpy (bdc—1,4-benzenedicarboxylate, bpy—4,4′-bipyridine) nanometer sized crystals^79^. Most guest-induced transformations in porous frameworks show a pronounced hysteresis in the adsorption isotherm, indicating the opening or closing process is an activated process.

During gate opening (case 1) the high activation barrier arises mostly from the hindered guest access into the pore. The gate opening transformation is an activated process, which requires a high “overpressure” (Δ*p*) for the guests to intrude into a formally inaccessible pore space, while during closing, the barrier for desorption is small, and hence closing occurs close to equilibrium^81^. Additional activation barriers arise from the solid–solid transformation where a phase transition, in many cases causing changes in cell volume and symmetry, is associated with grain boundary formation or even cracking. Kinetics of nucleation are mostly governed by heterogeneous nucleation theory. Pore opening is associated with a fractional volume expansion *δν/ν* of the nucleus, which deforms the closed phase (matrix) surrounding the nucleus, significantly enhancing the overall activation barrier^82^:

2 $$\Delta G = \frac{4}{3}\pi R^3\Delta G_V + \frac{2}{3}\mu \frac{{\left( {\delta \nu } \right)^2}}{\nu }E\left( {y/R} \right) + 4\pi R^2\sigma$$

This elastic contribution scales with the matrix shear modulus *µ* and the fractional volume change *δν/ν*. In principal both quantities can be calculated using simulation tools^63,67,83^. Volume changes can be estimated by analyzing the Helmholtz free-energy of the host with respect to the cell volume and subsequent identification of the minima. A bistable hinge, for example, a cluster-bound carboxylate, with wide tolerance for deformation and consequently large fractional volume expansion (*δν/ν*) will cause an increased nucleation barrier. The same is true for a wide-bending linker. In contrast, small deformations (and small cell volume changes) are associated with low nucleation barriers and, in the limiting case, a nucleation-free continuous, second-order transition may be observed. As this nucleating phase has to work against the surrounding (yet untransformed) framework matrix its shear modulus, *µ*, plays an equally important role. In particular, densely packed and highly ordered molecular subunits adhering to each other via secondary interactions (dispersive, dipole etc.), but also rigid deformation potentials of the framework hinges, enhance the shear modulus and thus increase the nucleation barrier. *E(y/R)* is a dimensionless number characterizing the ellipsoidal shape of the nucleating phase ranging from 0 for flat plates to 1 for a sphere. Hence, flat nucleation is faster as proposed by Coudert for MIL-53 (Case 2a)^84^. In a qualitative sense, the identification of crystallographic directions of low shear moduli hints towards preferred transformation mechanisms associated with reduced nucleation barriers. The entire molecular simulation of nucleation phenomena in phase transitions is still challenging but significant methodological advances at least for molecular crystals have been made^85^. In this context the role of particle size and morphology becomes important, as observed for ZIF-8 by Lively and Watanabe^86–88^. While only minor structural (volume) changes accompany the ZIF-8 structural transformation, in DUT-8(Ni) a volume expansion, during gating, of more than 240% causes more drastic particle size effects: (a) repeated switching stiffens the surrounding matrix (*µ*), which shifts the gate-opening pressure to higher *p/p*_*0*_^89^; (b) at intermediate particle sizes (500 nm), guest-induced opening is completely absent^90^; and (c) below 500 nm the empty *op* phase is stabilized as a metastable phase^91^. Apparently, this barrier enhancement for dynamics resulting in rigidification of dynamic frameworks upon downsizing is a more general phenomenon than initially expected^66,79,80,86,89,91,92^. However, a theoretical framework explaining the textural origin of this rigidification is at a very early stage^86,93–95^. Hypotheses for these enhanced barriers in downsized dynamic frameworks encompass contributions from (1) interfacial energy (surface energy, matrix effects, surface deformation, reduced density of the adsorbed phase in vicinity of the surface) as well as (2) cooperative effects (ferroic coupling effects, defects, twin boundaries, grain boundaries, suppressed nucleation etc.). Despite lacking comprehensive theory, the observations clearly demonstrate tailorable activation barriers by size and shape, but these are only indirect observations of variable transformation rates and *t*-axis design.

In mesoporous materials, the nucleation of the fluid phase is also an activated process with its own temperature and pressure dependence leading to a hysteresis even without pronounced structural transformation^96–99^. The coupling of switchability and mesoporosity in a framework interlinks hitherto independent metastable phases leading to complex physical processes. We observed negative gas adsorption (NGA) for the first time in DUT-49, a mesoporous MOF showing colossal collective breathing motions induced by methane, butane and other gases (case 2b). NGA relates the pushout of molecules (Δ*n*) from a long-lived metastable (*op*) state to a high activation barrier for the colossal contraction of the MOF (*E*_*a,solid*_) and a barrier of pore filling (*E*_*a,fluid*_). In NGA materials, Δ*n* is an indirect measure for the kinetic hindrance of the transformation (i.e., the barrier *E*_*a*_ or temporal evolution). For a given adsorptive and temperature, Δ*n* serves as a descriptor for *E*_*a,solid*_ and hence identifies a characteristic temporal evolution of a crystal. Tailoring the crystal size was shown to tune Δ*n* for DUT-49 and a given adsorptive, in a wide range^66^. This is what we call *t*-axis design.

However, further development is needed for direct observation of framework dynamics as a response to molecular stimuli in situ.

## Challenges and advanced methods for analyzing a framework-intrinsic *t*-axis

**Fig. 5:**
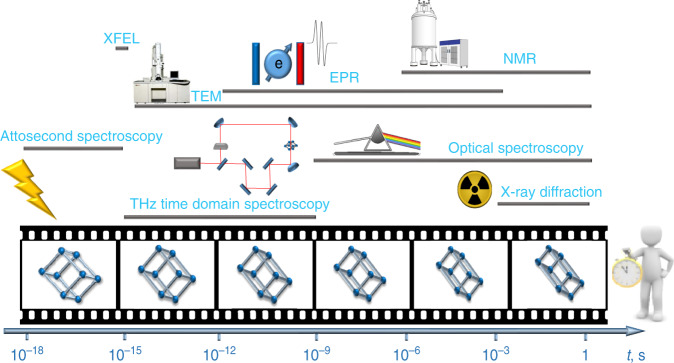
Time-resolved analytical in situ methodologies. Future development of advanced complementary tools is essential to assess dynamic transformations over diverse timescales.

The development of time-resolved operando techniques is highly advanced in the field of catalysis and biological systems^74,100,101^. Dynamic frameworks, discussed here, typically respond via cooperative mechanisms to subtle pressure changes in the gas phase, resulting in pronounced structural transformations. Gas adsorption, alone, can reveal the presence of metastable states^65,66^ but this kinetic information is acquired for an ensemble of crystals potentially switching with different rates. The quest for understanding specific host-guest-interactions in crystalline framework materials has significantly stimulated the rise of specifically adapted and parallelized characterization techniques (Fig. 5)^102–106^, as structural intermediates are only observable in situ, i.e., at a specific loading of the guest, by controlling the activity, temperature or external pressure. The analysis of elastic deformations and local interactions by inelastic neutron scattering^30,107^, NMR^13,108^, IR, and Raman spectroscopy^13^ in the presence of guest molecules is well established^109,110^. Solid-state NMR provides important insights into intrinsic dynamics of solids. In particular, deuterium NMR is established for molecular motions^111,112^ but is not applicable to inelastic deformations. NMR spectroscopy may achieve 10^−6^ s time resolution, and ^2^H NMR is a powerful technique for analysing molecular rotors and other molecular motions^113^. In terms of fluid phase analyses, pulsed field gradient nuclear magnetic resonance (PFG NMR) is a widely established method for the analysis of guest motion in pores^114^. PFG NMR can be used to analyse a wide range of diffusion rates in crystals^115^, however, this technique has never been applied to analyze a dynamic pore opening process in a switchable MOF.

The analysis of inelastic cooperative molecular framework dynamics on the nano- and picosecond timescale clearly requires new in situ instrumentation. A customized environment for capturing the spatiotemporal evolution of 4D-MOFs is yet to be invented. Direct measurement of the temporal evolution of dynamic MOFs is an open quest. The transformation rate for inelastic deformations may vary over several orders of magnitudes, as it scales exponentially with Δ*T* and Δ*p*. Only a few degrees of undercooling can enhance the nucleation rate by a factor of 100. As phonons, grain boundaries may propagate with rates >10^9^ Hz through the crystal, dynamic frameworks require advanced time-resolved experimentation. This instrumentation is established for light-induced processes using pulsed photon sources. Recent development of serial protein crystallography at X-ray Free Electron Laser facilities can reach time resolution down to pulse lengths of 10^−15^ s^116–118^. However, guest-induced transformations require a step-wise increase in pressure or concentration at least one order of magnitude faster than the characteristic time constant for the dynamic transformation. Otherwise, the intrinsic *t*-axis of the solid is obscured by the superimposed kinetics of the gas-pressure increase around an individual crystal (controlled by convection and diffusion in the embedding reservoir). Optical spectroscopy is useful in analysing a wide range of timescales but requires an optical probe in the framework. Only in photoswitchable frameworks can this wide range of existing time-resolved optical techniques be applied. However, a molecular stimulus is the dominant source of dynamics in switchable frameworks and thus the coupled kinetics of guest movement and framework response require novel analysis techniques.

Time-resolved powder diffraction only reaches orders of 10^-3^ s, and intrinsic limitations exist in terms of instant sample exposure to a gas-pressure stimulus. On the other hand, the development of pressure pulse methods in combination with high temporal resolution structural methods would provide deep insights into truly 4D materials^118^. The recent development of in situ electron microscopy methods, in gas and fluid environments, is rather promising for developing the field. Electron diffraction using pulsed lasers may give access for a wide range of temporal resolution required to analyse transformations at GHz, or even faster rates at the level of individual crystals. Electron diffraction has reached timescales down to 10^−13^ s^119^. However, available stimuli are limited and gas and liquid dosing is in an early development phase for TEM technologies.

The development of temporally resolved analytic techniques, adapted to capture dynamic pore opening of switchable frameworks, remains a key task to achieve an understanding of important factors affecting the temporal evolution of dynamic MOFs. Exact analysis of their transformation rates is the foundation for the deliberate tuning of the 4th dimension.

The predictive framework for dynamic solids has made significant progress in recent years^59,120,121^. A few approaches for activation energy estimation are already reported^81^. However, the theoretical understanding of particle size effects, cooperativity, role of outer surface, and defects on switching kinetics and spatiotemporal evolution is at an early stage.

## The design of 4D frameworks toward deliberate tuning of the temporal dimension

**Fig. 6:**
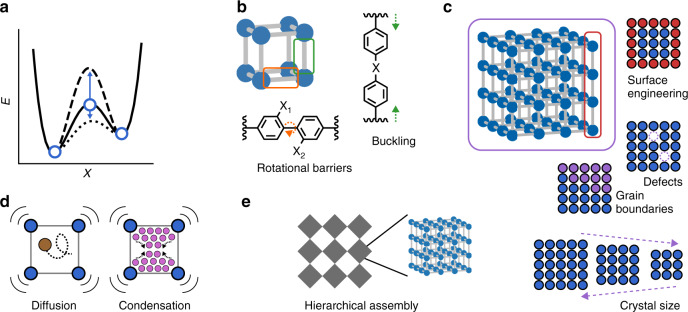
Measures for tailoring activation barriers and thus temporal evolution in porous frameworks. **a** diagram of the activation barrier with respect to a framework property, *X*. **b** molecular design. **c** cooperative effects. **d** coupling between the fluid and host. **e** hierarchical assemblies.

The outlined indirect observations of transformation barriers indicate plentiful opportunities for deliberate tuning of timescales in dynamic MOFs (Fig. 6).

### Molecular framework building blocks

The deliberate design of 4D frameworks is in its infancy. Size and shape tailoring of barriers (Δ*n,* Δ*p*) is barely beginning. Advanced “*t*-axis design” relies on molecular controlled assembly in 3D architectures. A first approach will focus on tuning buckling or rotational barriers in a single linker as a constituent of a network^67^. The bistability of frameworks is dominated by the deformability of their constituents, i.e., clusters and organic linkers. The potential surface characterizing molecular, cluster, or hinge deformation in a first approximation will significantly affect the spatiotemporal activation barriers. For example, a stiff linker or hinge will raise the activation energy and hence lead to kinetically hindered switching.

More complex situations will arise from torsional barriers or molecular interlocking. A second generation of *t*-axis designed frameworks may also take into account a rationale for collective barriers, such as coupling between unit cells. In principle, it should be possible to rationalize the deformation coupling between linkers of adjacent unit cells. Domino-like architectures can be envisioned in which the coupling and spatial organization of the “dominoes” (linkers, clusters, hinges) is well defined. Recent progress in the design of multivariate MOFs^54^ even indicates the future possibility of arranging a variety of linkers in frameworks in a sequential fashion, an important basis for *t*-axis design of dynamic systems in future. PolyMOFs are characterized by covalent bridges in between linkers defining the coupling strength^122,123^. Interweaving^124^ would provide a strong coupling scheme for linkers or even secondary covalent bonds (as for polyMOFs) could be introduced to deliberately engineer the propagation of buckling cascades in 4D-MOFs (“domino effect”). In nature these couplings are mainly realized through hydrogen bonds. The pioneering work by Rosseinsky supports the potential of hydrogen bonded dynamic frameworks with all implications^49^. As pointed out, the potential landscape of such materials is much more complex as compared to the simple dynamic systems discussed above. Instead of a few local minima they exhibit countably many minima, which change in relative energy depending on the guest present. These frameworks open huge opportunities to achieve high selectivity in recognition for more complex substrates by subtle tuning of energy barriers. Despite complex and unrealized theoretical modelling of these interactions, rationalization of the dynamics in such solids could lead to important applications of 4D materials in separation, catalytic conversion, sensing and biological applications. The synthetic “domino framework architectures” would certainly profit from computational in silico design. NMR could give important insights into noncovalent linker interactions, leading to coupled buckling motions and their time-scales. Currently, a comparison with DNA would overstate the actual synthetic and analytic abilities available. However, in principle, molecular framework architectures (MOFs, COFs etc.) pose the potential for encoding a higher level of complex information with an intrinsic linkage to its dynamic properties. The integration of a complex “time gene” that programs more complex motions, as postulated here, in a 4D framework remains a long-term vision.

### Cooperative effects and defects

The importance of size effects for switchable solids has been discussed^66,86,87,90^. Further development of size and shape engineering may be achieved by colloidal techniques, using microreactors, surfactant assisted syntheses etc. Recent literature gives a wide range of established synthesis techniques for MOF nanoparticles with more or less controlled morphology^125^. Engineering the surface structure by controlling the termination chemistry, surface functional groups, coatings etc. could provide further means of controlling the *t*-axis. Controlling defect type and concentration, on the other hand, poses massive challenges. In particular, the role of grain boundaries requires improved understanding and analytic insights into factors affecting the number and type of intrinsic grain boundaries. They may accelerate kinetics serving as nucleation centers, and entrance channels for the guest, but also decelerate the phase transition if the propagation of the transformation through the crystal is scattered.

### Coupling of the molecular and solid host time-axis

The movement and adsorption of molecules in rigid porous materials, as a function of temperature and pore size, is relatively well understood. However, the coupling of diffusion to cooperative phase transformations remains unexplored, providing an opportunity for tuning timescales by adjusting the molecular diameter, interactions, temperature, and pressure (or concentration). A non-porous framework opens its pores for a guest molecule simultaneously diffusing into the emerging channel. It is questionable if a separate treatment of fluid and solid phase will ever lead to a profound understanding as cause and consequence are highly intermingled, characteristic of a coupled phenomenon. Temperature dependence of characteristic time and length scales are highly non-linear and dissimilar for the framework solid and guest fluid. In particular, for the solid, cooperative movements determine the energetics and barriers involving microscopic and macroscopic displacements initiated by an external stimulus. In contrast, the characteristic critical quantities of the fluid guest, such as critical temperature and pressure, are mostly dominated by molecular characteristics and near neighbour interaction potentials, without long range coupling effects determining diffusion rate constants. It is certainly the coupling of these dissimilar phases, which will lead to complex and novel kinetic phenomena. In praxis, the wide range of gases or solutes available will enable wide reaching control of the actuating rate of the framework, from slow to fast, depending on the kinetic diameter and adsorption enthalpy of the guest molecule.

### Hierarchical assembly and metamaterials

The application of dynamic phenomena into actuators^50^ and sensors^40,41^ requires their integration into macroscopic devices (films, monoliths, shaped bodies etc.). The few reports available, interestingly, pay little attention to the intrinsic kinetics of the devices^126^. The rational arrangement of dynamic MOFs into hierarchical architectures has tremendous potential. Firstly, the access channel dimensions will control the mass transport rates for stimuli inducing gases. Secondly, the positioning of dynamic materials in specific positions may create more complex machines, with responsive artificial knees and active hinges. Combining these prospects with the potential of emerging 3D printing technology^127^ represents a tremendous opportunity for the development of robots, self-unfolding, and self-sustaining systems^128^.

For macroscopic objects, like actuators or self-folding devices, hierarchical pore systems are essential to provide access for stimuli inducing fluids that will determine the kinetics of movements. A non-hierarchical system with uniform micropore size will have inherent transport limitations. On the other hand, with 3D printing it is envisioned that macro-sized channels will guide the guest molecules rapidly to the actuating MOF voxels in a 3D architecture. Hence, the hierarchical architecture can control the 4th dimension, time, to a certain degree but also the directions of complex motions by predefined gradients for self-movement of the system. These systems may be valuable constituents of medical implants, threshold switches, or self-sustaining robots for operation in harsh environments in future.

## Language and prediction

The chameleon-like structural change over time implies that dynamic materials “remember” a characteristic history. The development of adequate terminology is hence a key target to rationalise further development. In essence, the history should be included in the chemical name, like a sequence in a genome. A language clarification reporting all states and intermediates the material has “recorded” would significantly improve the reproducibility of dynamic phenomena reported in literature. The traditional use of materials descriptors is static and focussed on chemical composition and crystal structure. Clearly, this is not sufficient for responsive materials undergoing complex spatiotemporal changes during desolvation and repeated pore size changes. A dynamic framework records repeated structural transformational changes as an imprinted “history” in its microstructure in the form of twin- and grain boundaries and defects, which are typically not associated with significant compositional changes. In its simplest form a string of letters attached to the framework name (DUT-49_ABC…) may represent the history of such a framework, where A, B, C etc. represent different step-wise transformations of the material. However, the latter is a stark simplification, as the real trajectories of framework dynamics are not simple steps but may have more complex spatiotemporal character. Digitalization will play an important part in future as it may store a more complex trajectory belonging to a material and its characteristics.

The implications for scientific communication and publications are obvious. Not only does a rigorous description of the history of these materials have to be established. It may be even more important to develop scientifically adequate data representation and handling. For future publications, there are significant challenges associated with the sheer magnitude of time-resolved data and the visualization, and documentation of complex dynamic movements along the fourth dimension.

## Summary

Engineering the spatiotemporal evolution of dynamic frameworks is within reach. This is what we call *t*-axis design or deliberate engineering of 4D open frameworks. A stimulus initiates the cooperative microscopic motions of the framework, while the subsequent temporal evolution is self-controlled by system-intrinsic activation barriers that can be deliberately integrated into a 3D architecture using synthetic strategies, such as building-block assembly, interlocking, weaving, size control, defect engineering, etc. established for porous framework architectures over recent decades. Advancing the understanding and tunability of activation barriers and time-constants for transformable MOFs requires interdisciplinary efforts in chemistry, physics, instrumental development, computational science, and engineering. The required deliberate tuning of activation barriers is in its infancy. In particular, the interplay of molecular, cooperative and nucleation activation barriers in porous switchable solids and their interactions with a dissimilar fluid-like guest phase requires significant experimental and theoretical developments, in a wide range of time- and length scales. Further development of time-resolved analytical in situ instrumentation is essential for any progress in this field.

The macroscopic movements of 4D frameworks may pave the way to rate-controlled actuators, dampers, and self-sustaining medicinal implants. A temporal response poses huge potential in developing more complex time-dependent functionality, including periodic, accelerated, or self-terminated processes. Deliberate engineering of activation barriers in porous frameworks, interacting with molecular guest species, ideally guided by computational methods, will lead to enzyme-like catalysts changing their pore size dynamically for the desired substrate or expelling the desired product by pore closing. Kinetically controlled recognition of molecules by 4D frameworks may have important implications for energy efficient separations, biomolecule separation, sensing, and membrane development.

Changing the current view of materials science towards a time-dependent perspective may open new horizons, a paradigm shift for advanced materials design.
